# Effects of the beta-blocker carvedilol on arrhythmia and long-term clinical outcomes in benign prostate hypertrophy patients

**DOI:** 10.1097/MD.0000000000035008

**Published:** 2023-09-08

**Authors:** Soo Jin Kim, Han Su Park, Pil Moon Kang, Bong Joon Kim, Hyun Su Kim, Jung Ho Heo, Taek Sang Kim, Sung Il Im

**Affiliations:** a Division of Cardiology, Department of Internal Medicine, Kosin University Gospel Hospital, Kosin University College of Medicine, Busan, Republic of Korea; b Kang Pil Urology Clinic, Busan, Republic of Korea; c Department of Urology, Kosin University Gospel Hospital, Kosin University College of Medicine, Busan, Republic of Korea.

**Keywords:** arrhythmia, benign prostate hypertrophy, beta-blocker, carvedilol, clinical outcomes

## Abstract

Benign prostatic hypertrophy (BPH) is associated with autonomic dysfunction and sympathetic nervous system mediated by the alpha receptor. However, limited data exist regarding the effects of the beta-blocker (BB) carvedilol on arrhythmia and urologic outcomes in BPH patients. Our database of patients diagnosed with BPH from 2015 to 2020 was used to obtain echocardiography and electrocardiogram data. Inclusion criteria were BPH patients taking BBs. International Prostate Symptom Score questionnaire were used to evaluate the urinary symptoms and quality of life. Among 448 patients with BPH (69.2 ± 10.9 years) taking BBs, 219 patients took carvedilol (48.9%) and 229 patients took a non-carvedilol BB (51.1%; bisoprolol, 184 patients, 80% or nebivolol, 45 patients, 20%). Difference in the baseline characteristics was not observed. During the median 36-month follow-up, a lower incidence of arrhythmic events (*P* = .029), total urologic events (*P* < .001), and less use of additive alpha-blocker was observed in the carvedilol group (*P* = .022). In multivariate analysis, less carvedilol use (*P* = .019), heart failure (*P* < .001), stroke (*P* < .001), and cardiomyopathy (*P* = .046) were independent risk factors for arrhythmic events. In addition, less carvedilol use (*P* = .009) and older age (*P* = .005) were independent risk factors for urologic events based on BB type at the median 36-month follow-up. The use of carvedilol was associated with less arrhythmic events in BPH patients with palpitation and decreased the incidence of urologic events in BPH compared with the use of non-carvedilol BBs in long-term follow-up.

## 1. Introduction

Benign prostatic hyperplasia (BPH) is a common disease affecting middle-aged and elderly males. The disease may affect up to 50% of males 60 to 69 years of age and its prevalence increases with age. Individual symptoms of BPH may vary, although the condition is chronic and can significantly influence quality of life. Although lower urinary tract symptoms are common urologic problems, the pathophysiologic changes in urinary tissue are not fully understood. Known factors in the pathophysiology of lower urinary tract symptoms-related BPH include intrapelvic pressure elevation, vasoactive, and inflammatory mediators such as activation of the renin-angiotensin system, expression of transforming growth factor alpha, endothelial growth factor, and prostaglandin.^[1]^ Recently, reactive oxygen species, which are formed during ureteral obstruction, have been suggested to play a role in this process.^[2]^

Increasing evidence links inflammation to a broad spectrum of cardiovascular conditions such as coronary artery disease. In addition, emerging data support the association between inflammation and arrhythmia including atrial fibrillation (AF), the most common clinically significant arrhythmia in clinical practice.^[3–5]^ AF is often associated with other cardiovascular diseases including diabetes, hypertension (HTN), heart failure (CHF), ischemic heart disease, valvular heart diseases, and other cardiomyopathies. In 10% to 15% of the cases, AF occurs in the absence of any such comorbidities and is considered lone AF.^[3]^ However, in recent studies, other factors having a role in the genesis of AF have gained attention including obesity, sleep apnea, alcohol abuse and other intoxications, exercise, latent HTN, genetic factors, acid reflux disease, and local or systemic inflammation.^[4–6]^

Carvedilol is a nonselective beta-blocker (BB) with additional alpha-blocker properties. Carvedilol competitively blocks alpha-1, beta-1, and beta-2 adrenergic receptors, and has vasodilatory properties. Carvedilol has been commonly used for the clinical treatment of HF, HTN, and myocardial infarction. Carvedilol has antioxidant properties in addition to antiadrenergic effects. Due to its antioxidant properties, carvedilol can treat many pathologic conditions associated with enhanced cellular oxidative stress.^[6,7]^

However, data regarding the effect of carvedilol on the long-term clinical outcomes in BPH patients are limited. In the present study, the effects of carvedilol on the long-term urologic and arrhythmic events in BPH patients with palpitation were evaluated.

## 2. Methods

### 2.1. Study population

In the present study, the medical records of 832 patients diagnosed with BPH at Kosin University Gospel Hospital from April, 2015 to December, 2020 were retrospectively reviewed.

Inclusion criterion was BPH patients diagnosed with ICD code (N40, ICD 10, 9) who received BB. Exclusion criteria included patients who took 5-alpha reductase inhibitor (PROSCAR) before carvedilol, patients lost to follow-up, history of valvular or congenital heart disease, hepatic or renal disease (known chronic liver disease, stage 3 advanced chronic kidney disease), acute cardiovascular or cerebrovascular event within the preceding 3 months, major trauma or surgery within the preceding 3 months, hyperthyroidism, or uncontrolled HTN. In addition, the following disease-specific exclusion criteria for prostate disease were used: previous prostatic surgery, urinary symptoms caused by conditions other than BPH, prostatic malignancy or a prostate-specific antigen level > 4 ng/mL, or both, and persistent postvoid residual urine volume (PVR) > 200 mL.

Finally, 448 consecutive BPH patients (mean age, 69.2 ± 10.9 years) were enrolled in the study. All patients were monitored to evaluate arrhythmic events and urologic parameters including total International Prostate Symptom Score (IPSS), voiding volume, voiding volume, maximum urinary flow rate (Qmax), IPSS change, IPSS storage change, Qmax change, incidence of additive prescription of alpha-blocker or anticholinergics, and urologic events^[8,9]^ that were defined including frequency, urgency, weak stream, nocturia, surgical, and interventional procedures for BPH based on the use of carvedilol during follow-up.

### 2.2. Ethical considerations

The study protocol conforms to the ethical guidelines of the 1975 Declaration of Helsinki, and the research protocol was approved by the ethics committee of Kosin University Gospel Hospital (IRB no. 2015-11-010). All patients provided written informed consent.

### 2.3. Data collection

After electrocardiogram (ECG) and chest X-ray, cardiovascular status was evaluated for each patient using echocardiography, an exercise test, 24-hour Holter recordings, and blood laboratory data from the initial visit, as determined by the attending physicians. From the database, the following information was collected: Patient data, including sex, age, height, and weight; Cardiovascular risk factors, including HTN (use of antihypertensive agents, systolic blood pressure ≥ 140 mm Hg, or diastolic blood pressure 90 mm Hg on admission) and diabetes mellitus (use of oral hypoglycemic agents or insulin, or glycosylated hemoglobin ≥ 6.5%); Cardiovascular disease status, including structural heart disease, congestive CHF, or a history of a disabling cerebral infarction or transient ischemic attack; Use of medication. Body mass index was calculated as weight in kilograms divided by the square of height in meters (kg/m^2^).

### 2.4. Definition of atrial and ventricular arrhythmia

In the present study, paroxysmal AF at the initial visit was defined as sinus rhythm on ECG and previous diagnosis of paroxysmal AF by the referring physicians. Patients whose AF was estimated to persist for ≥ 7 days after the initial visit were considered to have persistent AF and were excluded from the analysis. During the follow-up period, the onset of persistent AF was defined as the first time in which all ECGs indicated AF after ≥ 3 consecutive ECGs at intervals of ≥ 1 week after the initial examination, and chronic AF was defined as AF present for at least 6 months without intervening spontaneous episodes of sinus rhythm for which cardioversion was unsuccessful and subsequently not attempted.^[10]^ When 3 ECGs could not be obtained during the period, the physicians made a clinical judgment regarding the onset time of AF progression. Atrial arrhythmia during follow-up was defined as atrial premature complex, atrial tachycardia, or atrial flutter. Ventricular arrhythmia during follow-up was defined as ventricular premature complex, ventricular tachycardia, or ventricular fibrillation.

### 2.5. Definitions of urodynamic parameters

Efficacy evaluations for urodynamic parameters included Qmax and Qavg urinary flow rates as well as PVR. Uroflowmetry was performed with a Flomex PW24 flowmeter (JEPAL, Bialystok, Poland). The IPSS questionnaire was used to determine the severity and bother scores of BPH symptoms in patients. Study medication compliance and concurrent medications were evaluated. Adverse events were recorded and monitored accordingly.

### 2.6. Clinical endpoints

The primary end points were arrhythmic events including atrial premature beat, atrial tachycardia, atrial fibrillation, ventricular premature beat, and ventricular tachycardia during follow-up. The secondary end points were defined as the change in PVR, Qmax, and IPSS, and total urologic events including frequency, urgency, weak stream, nocturia, urologic interventional procedure, medication changes, and rehospitalization.

### 2.7. Transthoracic echocardiography

All enrolled patients underwent 2-dimensional transthoracic echocardiography. The examinations were performed using a commercially available Vivid 7 (GE Medical System, Vingmed, Horten, Norway) ultrasound system. All recorded echocardiograms were measured and interpreted with clinical information blinded using a computerized off-line analysis station (Echopac 6.3.4; GE Medical System). All measurements were derived from 3 consecutive cardiac cycles and averaged. The left ventricular dimensions, wall thicknesses, and left atrial dimensions were determined in the parasternal long-axis view with the M-mode cursor positioned immediately beyond the mitral leaflet tips perpendicular to the long-axis of the ventricle according to the recommendations by the American Society of Echocardiography.^[11]^ The left ventricle ejection fraction was obtained via the modified biplane Simpson method from the apical 4- and 2-chamber views.

### 2.8. Statistical analysis

All continuous variables are expressed as either the mean ± standard deviation or median (25th, 75th interquartile range) depending on the distribution. For continuous data, statistical differences were evaluated using Student *t* test or the Mann–Whitney *U* test depending on the data distribution. Categorical variables are presented as frequencies (percent) and were analyzed using the chi-squared test. To determine whether any of the variables were independently related to arrhythmic and urologic events based on type of BBs, a multivariate analysis of variables with a *P* value < .05 in the univariate analysis was performed using linear logistic regression analysis. All correlations were calculated using Spearman rank correlation test. All statistical analyses were conducted using the SPSS statistical software, version 19.0 (SPSS Inc., Chicago, IL), and statistical significance was set at *P* < .05 (2-sided).

## 3. Results

The baseline demographics for both groups are listed in Table 1. In the present study, among the 448 BPH patients taking BBs, 219 patients used carvedilol and 229 patients used non-carvedilol BBs. Baseline characteristics were not statistically different in both groups.

**Table 1:** Baseline clinical characteristics in patients with BPH based on BB type.

Variables	Carvedilol group (n = 219)	Non-carvedilol group (n = 229)	*P* value
Age (yr)	69.3 ± 10.8	68.9 ± 10.9	.456
CHF (%)	33 (16.3)	28 (13.0)	.406
DM (%)	61 (27.8)	62 (27.4)	.122
HTN (%)	137 (67.2)	125 (58.1)	.069
CVA (%)	49 (24.4)	38 (17.4)	.092
CAD (%)	52 (25.9)	41 (19.0)	.100
CMP	7 (3.5)	11 (5.1)	.656
HCMP (%)	3 (1.5)	6 (2.8)	
DCMP (%)	4 (2.0)	5 (2.3)	
COPD (%)	10 (5.0)	5 (2.3)	.189
Alcohol (%)	60 (29.7)	57 (26.4)	.513
Smoking (%)	56 (27.7)	66 (30.6)	.591
CKD	21 (10.4)	13 (6.0)	.109
Urology test			
IPSS total	12.1 ± 8.0	13.1 ± 9.1	.345
IPSS storage	5.2 ± 3.9	5.6 ± 4.6	.477
IPSS voiding	6.6 ± 5.8	7.7 ± 6.2	.111
IPSS QOL	3.3 ± 1.2	3.4 ± 2.1	.757
Qmax	12.9 ± 9.3	12.5 ± 8.2	.837
Prostate size (gm)	36.0 ± 15.5	35.3 ± 17.1	.129
Laboratory findings			
WBC (10^3^/uL)	8.3 ± 3.7	9.0 ± 1.3	.432
Bilirubin (mg/dL)	0.9 ± 0.4	0.9 ± 0.4	.862
AST (mg/dL)	32.5 ± 22.8	32.7 ± 14.2	.975
ALT (mg/dL)	31.0 ± 19.8	28.4 ± 22.0	.645
Glucose (mg/dL)	106.8 ± 78.6	95.7 ± 63.4	.128
Total cholesterol (mg/dL)	158.7 ± 50.1	166.6 ± 51.2	.115
LDL (mg/dL)	89.8 ± 45.0	94.5 ± 46.2	.300
Triglyceride (mg/dL)	121.4 ± 82.0	124.6 ± 75.8	.681
Creatinine (mg/dL)	1.4 ± 1.3	1.2 ± 0.8	.125
hsCRP (mg/dL)	1.8 ± 0.5	1.4 ± 0.4	.277
PSA (ng/mL)	3.5 ± 1.2	3.3 ± 0.9	.239
Echocardiogram parameters			
LVEF (%)	65.3 ± 46.0	63.8 ± 10.6	.662
LVID (mm)	32.4 ± 12.4	33.3 ± 26.8	.662
LVIDd (mm)	48.0 ± 6.4	48.1 ± 6.1	.898
IVSD (mm)	13.5 ± 3.0	13.4 ± 4.1	.822
LVPWD (mm)	11.3 ± 2.1	11.8 ± 8.2	.363
LAVI (mL/m^2^)	22.5 ± 12.3	24.2 ± 15.5	.633
E/A	3.8 ± 0.2	3.7 ± 0.6	.962
E/E’	11.9 ± 4.9	11.2 ± 6.8	.253
Medication			
Propafenone (%)	1 (0.5)	0 (0)	.492
Digoxin (%)	12 (5.6)	13 (5.8)	1.000
CCB (%)	63 (28.8)	50 (27.5)	.365
ARB or ACEi (%)	55 (25.5)	43 (23.5)	.407
Statins (%)	1 (0.5)	1 (0.4)	1.000
Aspirin (%)	78 (36.1)	82 (36.6)	.921
Alpha-blocker	193 (88.5)	154 (84.4)	.104
Anticholinergics	41 (19.0)	39 (21.2)	.322

Values are mean ± SD (range). E, the peak mitral flow velocity of the early rapid filling wave; A, peak velocity of the late filling wave due to atrial contraction; E’, early diastolic mitral annulus velocity; A’, late diastolic mitral annulus velocity.

ACEi = angiotensin converting enzyme inhibitor, ALT = alanine aminotransferase, ARB = angiotensin II receptor blocker, AST = aspartate aminotransferase, BB = beta-blocker, BPH = benign prostatic hypertrophy, CAD = coronary artery disease, CCB = calcium channel blocker, CHF = congestive heart failure, CKD = chronic kidney disease, CMP = cardiomyopathy, COPD = chronic obstructive pulmonary disease, CRP = C-reactive protein, CVA = cerebrovascular accident, DCMP = dilated cardiomyopathy, DM = diabetes mellitus, HCMP = hypertrophic cardiomyopathy, hsCRP = high sensitive C, HTN = hypertension, IPSS = International Prostate Symptom Score, LAVI = left atrial volume index, LDL = low-density lipoprotein, LVEF = left ventricular ejection fraction, LVID = left ventricular systolic diameter, LVIDd = left ventricular diastolic diameter, PSA = prostate-specific antigen, QOL = quality of life; Qmax, maximum urinary flow rate, SD = standard deviation, WBC = white blood cell count.

The baseline laboratory and echocardiographic findings did not differ between the groups (Table 1).

The clinical outcomes at the median 36-month follow-up are shown in Table 2. A lower incidence of arrhythmic events (*P* = .034), total urologic events (*P* < .001), and less use of an additive alpha-blocker were observed in the carvedilol group (*P* = .022). A higher IPSS voiding change (*P* = .001) and decreased Qmax (maximum urinary flow rate) change (*P* = .006) were observed in the non-carvedilol group.

**Table 2:** Clinical outcomes in patients with BPH based on BB type at the median 36-month follow-up.

Variables	Carvedilol group (n = 219)	Non-carvedilol group (n = 229)	*P* value
Median follow-up duration (mo)	35.2 (16.6–57.0)	36.9 (16.2–59.7)	.824
Total any events (%)	189 (93.6)	192 (91.0)	.362
readmission (%)	144 (71.3)	156 (71.6)	1.000
Arrhythmic events (%)	38 (18.8)	61 (28.0)	.029
AF, AT, or PAC (%)	36 (94.8)	57 (93.4)	
PVC or VT (%)	1 (2.6)	2 (3.3)	
PSVT (%)	1 (2.6)	2 (3.3)	
Total urologic events	16 (7.3)	32 (17.5)	<.001
Frequency	14 (87.6)	18 (56.2)	
Urgency	1 (6.2)	10 (31.2)	
Weak stream	1 (6.2)	2 (6.2)	
Nocturia	0 (0)	1 (3.1)	
Interventional procedures	0 (0)	1 (3.1)	
Medications for urinary symptoms			
Alpha-blocker	177 (80.8)	207 (90.4)	.004
Anticholinergics	36 (19.4)	41 (22.5)	.522
Follow-up urology test (total 110 patients)	Carvedilol group (n = 56)	Non-carvedilol group (n = 54)	
Post IPSS total	11.3 ± 8.1	11.7 ± 8.8	.840
Post IPSS voiding	6.7 ± 5.5	6.4 ± 5.8	.777
Post IPSS QOL	2.9 ± 1.3	2.6 ± 1.3	.298
Post voiding volume	159.6 ± 142.3	264.9 ± 165.4	.484
Post Qmax	27.3 ± 44.1	18.4 ± 10.2	.103
IPSS change	2.7 ± 1.3	2.7 ± 1.2	.993
IPSS voiding change	1.6 ± 0.8	5.6 ± 4.7	.001
IPSS QOL change	0.9 ± 0.7	0.7 ± 0.2	.622
Qmax change	5.4 ± 5.5	−6.1 ± 6.2	.006

Values are median with quartile or mean ± SD (range).

AF = atrial fibrillation, AT = atrial tachycardia, BB = beta-blocker, BPH = benign prostatic hypertrophy, IPSS = International Prostate Symptom Score, PAC = premature atrial contraction, PSVT = paroxysmal supraventricular tachycardia, PVC = premature ventricular contraction, QOL = quality of life, Qmax = maximum urinary flow rate, VT = ventricular tachycardia.

In univariate analysis for arrhythmic events, carvedilol use, nocturia, CHF, stroke, and cardiomyopathy (CMP) were significantly associated with arrhythmic events including atrial premature beat, atrial tachycardia, atrial fibrillation, ventricular premature beat, and ventricular tachycardia in patients with BPH. In multivariate analysis, less carvedilol use (*P* = .019), CHF (*P* < .001), stroke (*P* < .001), and CMP (*P* = .046) were independent risk factors for arrhythmic events at the median 36-month follow-up (Table 3A).

**Table 3:** Univariate and multivariate Cox analyses for arrhythmic events including premature atrial contraction, atrial tachycardia, AF, premature ventricular contraction, and ventricular tachycardia (A), and for urologic events including frequency, urgency, weak stream, nocturia, and surgical and interventional procedures for BPH based on BB type at the median 36-month follow-up (B).

(A)	Univariate analysis		Multivariate analysis	
Variable, n (%)	OR (95% CI)	*P* value	OR (95% CI)	*P* value
Use of carvedilol*	0.596 (0.376–0.945)	.028	0.439 (0.221–0.873)	.019
Nocturia	3.418 (2.137–5.469)	<.001		
CHF	3.430 (1.945–6.051)	<.001	3.586 (1.922–6.691)	<.001
Stroke	1.767 (1.046–3.299)	.047	5.328 (3.060–9.278)	<.001
CMP	4.389 (1.682–11.456)	.003	4.180 (1.451–12.034)	.046
(B)	Univariate analysis		Multivariate analysis	
Variable, n (%)	OR (95% CI)	*P* value	OR (95% CI)	*P* value
Use of Carvedilol†	0.264 (0.123–0.567)	0.001	0.315 (0.132–0.752)	0.009
Use of Anticholinergics	0.487 (0.231–0.925)	0.041		
PSA	1.014 (1.001–1.028)	0.048		
Age	1.042 (1.007–1.078)	0.018	1.069 (1.021–1.120)	0.005

AF = atrial fibrillation, BB = beta-blocker, BPH = benign prostatic hypertrophy, CI = confidence interval, CHF = congestive heart failure, CMP = cardiomyopathy, OR = odds ratio, PSA = prostate-specific antigen.

* Use of carvedilol compared with use of other BBs.

† Use of carvedilol compared with use of other BBs.

In univariate analysis for urologic events, carvedilol use, anticholinergic use, higher prostate-specific antigen, and older age were significantly associated with urologic events including frequency, urgency, weak stream, nocturia, and surgical and interventional procedures for BPH based on BB type. In multivariate analysis, less carvedilol use (*P* = .009) and older age (*P* = .005) were independent risk factors for urologic events including frequency, urgency, weak stream, nocturia, and surgical and interventional procedures for BPH based on BB type at the median 36-month follow-up (Table 3B).

**Figure 1.**
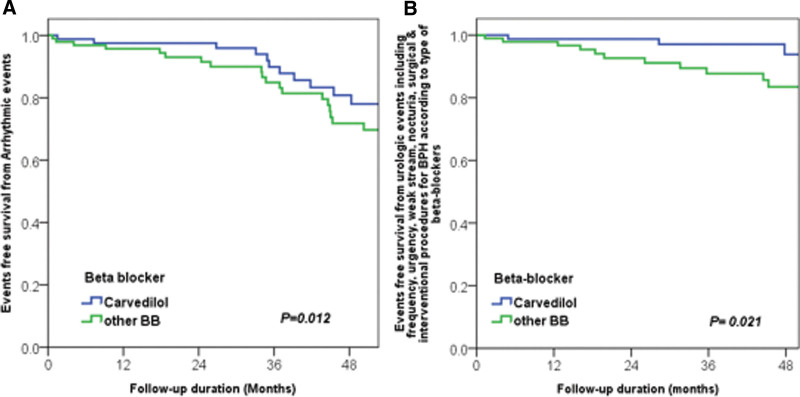
Kaplan–Meier analysis for event-free survival from (A) urologic events including frequency, urgency, weak stream, nocturia, and surgical and interventional procedures for benign prostatic hypertrophy (BPH), (B) arrhythmic events based on type of beta-blockers (BBs) in patients with BPH.

Kaplan–Meier analysis showed event-free survival from arrhythmic events (*P* = .012, Fig. 1A) and urologic events including frequency, urgency, weak stream, nocturia, and surgical and interventional procedures for BPH based on BB type (*P* = .021; Fig. 1B) was higher in patients using carvedilol compared with subjects using non-carvedilol BB at the median 36-month follow-up.

## 4. Discussion

In the present study, the use of carvedilol was associated with less arrhythmic events in BPH patients with palpitation. In addition, although the total IPSS did not differ between the groups, carvedilol improved the voiding volume and maximal voiding velocity and decreased the incidence of additive prescription of alpha-blocker and urologic events including frequency, urgency, weak stream, nocturia, and surgical and interventional procedures for BPH in the long-term follow-up.

In a previous study, BPH was reportedly associated with greater AF occurrence showing an approximate 1.19-fold increase in new AF in BPH patients.^[12]^ Specific situations such as inflammation, surgical stress, and other severe medical circumstances may function as “modulators” leading to arrhythmia including incident AF. The detailed pathophysiological mechanisms leading to the link between BPH and AF are difficult to determine due to the inherent nature of observational association studies. Notably, in that study, the surgical intervention for BPH contributed to reducing the risk of AF development.

Carvedilol is a lipophilic, third-generation, nonselective beta adrenoceptor and selective alpha-1 adrenoceptor blocker. In a previous study, alpha-1 and beta-1 adrenoceptor stimulation enhanced the automaticity through different mechanisms in the myocardium. Carvedilol, a dual antagonist of alpha and beta adrenoceptors was significantly more effective than bisoprolol, a selective antagonist of the beta adrenoceptor.^[13]^ Carvedilol also possesses significant antioxidant properties capable of scavenging free radicals as well as inhibiting reactive oxygen species production. In addition to the parent compound, its metabolites, SB 211475, BM 910228, and SB 209995, are powerful antioxidants and may contribute to the protective effect of carvedilol.^[14–16]^

Furthermore, inflammation was shown in numerous studies to possibly play a significant role in the initiation, maintenance, and perpetuation of arrhythmia including AF.^[17–21]^ In the present study, we hypothesized that carvedilol has antioxidant properties in addition to antiadrenergic effects and can treat many pathologic conditions associated with enhanced cellular oxidative stress due to its antioxidant properties. However, in the present study, significant difference was not observed in inflammatory markers such as white blood cell, high sensitive C, and neutrophil/lymphocyte ratio before/after BB treatment in both groups.

In previous studies, the association between BPH and risk of incident arrhythmia including AF was greater among subgroups of patients ≤ 64 years of age, those with comorbidities, and short follow-up period.^[12,22]^ In the present study, various factors were associated with arrhythmic events in patients with BPH, including carvedilol use, nocturia, CHF, stroke, and CMP. In multivariate analysis, carvedilol use, CHF, stroke, and CMP were independent predictors of arrhythmic events in patients with BPH at the median 36-month clinical follow-up (Table 3).

Selective alpha-1 adrenoceptor inhibitors were extensively used as first-line therapy for the treatment of HTN and coexisting BPH; therapy with the alpha-1-blocker doxazosin in males with HTN and cardiac risk factors was associated with a higher incidence of CHF compared with other antihypertensive agents.^[23]^ Consequently, the use of alpha-blockers to treat BPH may not necessarily result in the optimal management of concomitant HTN. These hypertensive patients may require separate therapy for high blood pressure. Therefore carvedilol, a BB with selective alpha adrenoceptor antagonist activity appears a reasonable alternative.^[24]^ In addition, the beta adrenoceptor subtype that mediates relaxation of the urinary bladder has been extensively studied and varies among mammalian species.^[25]^

This is the first study in which stratification was validated to assess the probability of arrhythmic events using carvedilol compared with non-carvedilol BBs and the effects of carvedilol evaluated on long-term urologic clinical prognosis in patients with BPH. Although the total IPSS (*P* = .345) did not differ between groups, the incidence of urologic events (*P* < .001) were lower in the carvedilol group than in the non-carvedilol BB group.

The results showed the use of carvedilol was associated with less arrhythmic events in BPH patients with palpitation and decreased the incidence of urologic events for BPH in the long-term follow-up. Although the mechanism remains unclear, the control of vagal tone increase associated with dysuria due to BPH using nonselective beta adrenoceptor and selective alpha-1 adrenoceptor blockers may reduce trigger activity and can be used as an important prevention tool for arrhythmia.

Further prospective studies are needed to determine if a true causal mechanism exists between the carvedilol use and arrhythmia as well as to determine whether the mechanism is dependent on a specific subtype of arrhythmia. In addition, the potential for carvedilol treatment to reduce the development of BPH-related urinary symptoms warrants further investigation.

The present study had several limitations. First, this was a single-center, retrospective study derived from a real-world practice with inherent limitations. Therefore, the study results should be considered as hypothesis-generating, and future prospective studies are warranted to confirm the results. Second, asymptomatic episodes of arrhythmia including AF may not have been recognized because arrhythmic events were based on clinical symptoms and ambulatory monitoring for a short period. Third, patients with potentially reversible causes were excluded from the study. Therefore, the results of this study cannot be extrapolated to other patient populations with previously detected paroxysmal tachyarrhythmia, such as paroxysmal AF. Fourth, the patients with BPH could not be continuously treated, therefore, a direct correlation of carvedilol treatment with urologic and arrhythmia-associated clinical outcomes could not be made. However, chronic inflammation and autonomic imbalance have been proposed as plausible pathophysiological mechanisms of urologic and arrhythmic events including AF. In addition, the results of this study have revealed a novel perspective for the use of carvedilol focusing on both the anti-arrhythmogenic effects and additive effects on BPH-related urologic symptom relief.

## 5. Conclusion

The use of carvedilol was associated with less arrhythmic events in BPH patients with palpitation and decreased the incidence of urologic events for BPH compared with the use of non-carvedilol BBs in long-term follow-up.

## Acknowledgements

We thank all members of the Division of Cardiology, Department of Internal Medicine, and Department of Urology, Kosin University Gospel Hospital for their assistance and support with data collection.

## Author contributions

**Conceptualization:** Sung Il Im.

**Data curation:** Soo Jin kim, Sung Il Im.

**Formal analysis:** Sung Il Im.

**Investigation:** Han Su Park.

**Methodology:** Bong Joon Kim, Hyun Su kim.

**Supervision:** Jung Ho Heo, Taek Sang Kim, Sung Il Im.

**Validation:** Pil Moon Kang, Sung Il Im.

**Visualization:** Sung Il Im.

**Writing – original draft:** Sung Il Im.

**Writing – review & editing:** Sung Il Im.
